# An evaluation of direct PCR amplification

**DOI:** 10.3325/cmj.2014.55.655

**Published:** 2014-12

**Authors:** Daniel E. Hall, Reena Roy

**Affiliations:** Forensic Science Program, Eberly College of Science, The Pennsylvania State University, University Park, PA, USA

## Abstract

**Aim:**

To generate complete DNA profiles from blood and saliva samples deposited on FTA® and non-FTA® paper substrates following a direct amplification protocol.

**Methods:**

Saliva samples from living donors and blood samples from deceased individuals were deposited on ten different FTA® and non-FTA® substrates. These ten paper substrates containing body fluids were kept at room temperature for varying lengths of time ranging from one day to approximately one year. For all assays in this research, 1.2 mm punches were collected from each substrate containing one type of body fluid and amplified with reagents provided in the nine commercial polymerase chain reaction (PCR) amplification kits. The substrates were not subjected to purification reagent or extraction buffer prior to amplification.

**Results:**

Success rates were calculated for all nine amplification kits and all ten substrates based on their ability to yield complete DNA profiles following a direct amplification protocol. Six out of the nine amplification kits, and four out of the ten paper substrates had the highest success rates overall.

**Conclusion:**

The data show that it is possible to generate complete DNA profiles following a direct amplification protocol using both standard (non-direct) and direct PCR amplification kits. The generation of complete DNA profiles appears to depend more on the success of the amplification kit rather than the than the FTA®- or non-FTA®-based substrates.

Direct amplification has gained increasing interest over the last few years. Newer, more robust amplification kits claim to perform direct amplification better and faster than kits previously available in the forensic science community. However, most of the commercially available amplification kits require pre-treatment of the body fluids with buffers or washing reagents prior to amplification. A primary advantage of direct amplification without purification of DNA is the reduced analysis time and higher throughput of databank samples.

Current methods for detection of short tandem repeat (STR) profiles from reference samples can employ direct polymerase chain reaction (PCR) amplification of body fluids bound to swabs, FTA® and/or non-FTA® (fast technology for analysis) substrates. Most collection media for storing dried body fluid samples contain cell-lysing chemicals to preserve DNA within a sample that may contain PCR inhibitors (1). Biological samples often contain PCR inhibitors such as proteins, lipids, polysaccharides and more, all of which can prevent direct PCR amplification (2). Devices such as the Buccal DNA Collector (Bode Technology Group, VA, USA) and the CEP® (Collect, Eject, Protect) Swab (Fitzco, Inc., MN, USA) do not contain lysing chemicals, and normally require an additional washing step. This procedure is usually necessary otherwise amplification quality may be poor and allele dropout may occur (3,4). These substrates containing body fluids must first be washed several times with purification reagents and extraction buffer. The process is time consuming and risks the possibility of sample contamination.

The aim of this research was to determine if current direct and standard amplification kits and substrates could produce concordant autosomal STR and Y-STR profiles. Another aim was to determine if body fluid punches from various areas of the same substrate yield profiles of similar quality as well as generate concordant DNA profiles.

## Materials and methods

Cleaning of laboratory surfaces and utensils with 10% bleach or 70% ethanol followed by a deionized water rinse, and frequent changing of gloves was routinely practiced to minimize contamination. A Harris Micro-Punch® 1.2 mm punching device (GE Healthcare Life Sciences, Little Chalfont, UK) was used to collect both blood and saliva samples. It should be noted that in the beginning of this research the Harris punching device was cleaned in between samplings. Later experiments indicated that this was unnecessary. Only aerosol resistant tips were used for pipetting.

### Collection media

The following ten collection media were used for this study: ProPRIMEi Indicating Micro, 705 Micro, Blood Direct Cards #1 and #2, Fitzco Collection Card, CEP® swab, ProPrime Direct (Fitzco, Inc.), Whatman EasiCollect, FTA® Indicating Micro (GE Healthcare Life Sciences), and the Buccal DNA Collector (Bode). Both the CEP® swab (Fitzco, Inc.) and the Buccal DNA Collector (Bode) are paper-based substrates that contain no cell lysing agents.

### Sample collection

Two blood samples were obtained from two deceased men by a pathologist prior to this study and kept frozen for approximately two years in collection tubes. Female blood was not collected for use due to limited availability of deceased blood samples. The blood collection tubes contained EDTA, a preservative and chelating agent used to prevent DNA degradation (5). Two saliva samples were collected from living female volunteers and kept frozen for one day to approximately one year prior to deposition on each substrate. One saliva sample collected previously from a living male donor and kept frozen for three years was used for STR and Y-STR analysis.

### PCR amplification kits

Body fluid punches were amplified with PowerPlex® 16, 16 HS, 18D, and 21 Systems from Promega Corporation (Fitchburg, Wisconsin, USA), and AmpFlSTR® Identifiler® Direct, Identifiler® Plus, and Identifiler® PCR Amplification Kits from Applied Biosystems (Foster City, California, USA). Similarly, 1.2 mm punches of the substrates containing male body fluids were amplified with PowerPlex® Y23 (Promega Corporation) and AmpFlSTR® Yfiler PCR Amplification Kit (Applied Biosystems).

### PCR amplification

Both blood and saliva samples were initially amplified following the manufacturer’s recommended thermal cycling protocols. At first, the recommended reaction volume (25 µL) was used for all samples. After it had been determined that complete DNA profiles could be obtained, the following attempt was made for the purpose of comparison and minimization of expenses in subsequent experiments. Selected substrates were subjected to amplification using half of the recommended reaction volume (12.5 µL). Half reaction volumes were amplified with AmpFlSTR® Identifiler® Direct, Identifiler® Plus, and Yfiler PCR Amplification Kits (Applied Biosystems). It was necessary to reduce cycle numbers from the recommended protocols when using reduced reaction volumes. Reducing reaction volumes adjusted the ratio of available template DNA to available deoxynucleotide triphosphates (dNTPs) in the reaction mixture, and corrected for high RFU (relative fluorescence unit) peaks and an increased presence of minus A peaks.

DNA from the body fluids was amplified using primers and reagents contained in each amplification kit. Amplification of autosomal and Y-STR loci was performed on a GeneAmp® 9700 Thermal Cycler (PE Applied Biosystems). The manufacturer’s recommended thermal cycling protocol was initially followed for each amplification kit. Deviation from the recommended thermal cycling parameters (ie, increasing/decreasing the number of PCR cycles) was only performed in cases where the presence of minus A peaks, stutter peaks, peak imbalance, allele dropout, etc. were noted.

### DNA extraction and quantification

DNA extraction and quantification were performed for use as supplemental procedures and were not used routinely as part of the direct amplification protocol. Each body fluid stained substrate was extracted and quantified twice for comparison purposes. Extraction and purification were performed with the Qiagen EZ1 DNA Investigator Kit (Qiagen, Hilden, Germany) (6). Quantification was performed using the Quantifiler® Human DNA Quantification Kit from Applied Biosystems (7).

### Capillary electrophoresis

The amplified products were analyzed via capillary electrophoresis using an AB 3130xl Genetic Analyzer (Applied Biosystems). The data were analyzed and alleles called using GeneMarker® HID Software Version 2.2 from SoftGenetics® (State College, PA, USA).

## Results

### Experimental design

All body fluid samples were immediately frozen after collection until deposited on the ten sample storage devices described above. Both saliva and blood samples were thawed at room temperature prior to deposition. Once thawed, 10 µL aliquots of blood and 20 µL aliquots of saliva samples were deposited with a pipet onto various areas of the ten substrates. A larger volume aliquot of saliva was used to increase the amount of nucleated cells available for amplification. After the body fluids were applied, all substrates were kept at room temperature ranging from 24 hours to approximately one year. Only single-source samples were used in this study. The study did not include mixtures of body fluids or mixtures of body fluids from two or more individuals.

A 1.2 mm punch containing one type of body fluid sample was taken at a minimum of 24 hours after deposition and added to a PCR tube with the appropriate PCR amplification reagent. Initially, one punch of a substrate containing a saliva sample was amplified. However, in later experiments two punches were amplified in order to increase the amount of template DNA available. Thermal cycling numbers remained the same for either one or two punches.

Punches were collected from various locations of the substrates from the area where the stains were deposited. This was performed in an attempt to determine if biasing was present due to uneven cell or chemical lysing agent distribution. The body fluid samples were each exposed to direct PCR amplification using the previously mentioned autosomal and Y-STR kits. The amplified products were analyzed following the appropriate manufacturer’s protocols, and peak amplitude thresholds were set at 50 RFU for all amplification systems. Alleles were assigned by comparison to the appropriate allelic ladder.

Generated DNA profiles from each individually sourced body fluid were compared for concordance to ensure all alleles were properly called and all profiles were complete. Profiles that do not fit these criteria were not considered concordant. The robustness of each PCR amplification kit was monitored based on calculated success and failure rates. A similar comparison of concordance and robustness was made with each substrate.

### DNA quantification

Prior to quantitating DNA from the punches of the body fluids deposited on each substrate, it was assumed that approximately 2-5 ng of template DNA was present in the 1.2 mm punches collected from blood (8). Twenty-six samples were randomly collected for quantitative analysis. Quantification was performed on at least one 1.2 mm punch of every substrate, but not necessarily for both body fluids for each substrate due to limited availability of blood samples. The data indicate that there may be some variability among the saliva samples’ quantity of DNA. This is likely due to the variable amount of nucleated cells present in a given saliva sample.

The average quantity of DNA in bloodstained punches was 3.32 ng. Fitzco’s ProPrime Direct card (Fitzco, Inc.) yielded the highest quantity of DNA per bloodstained punch at 3.39 ng. The substrate with the lowest quantity of DNA per bloodstained punch was ProPRIMEi Indicating Micro (Fitzco, Inc.) with a value of 3.23 ng. A second bloodstained punch collected from the ProPrime Direct (Fitzco, Inc.) was an outlier in the quantitative results with a value of 0.334 ng DNA, and was not included in the average calculation.

The average quantity of DNA in saliva-stained punches was 2.98 ng. Fitzco’s Blood Direct Card #2 (Fitzco, Inc.) yielded the highest quantity of DNA (3.28 ng) from the saliva-stained punch. The substrate with the lowest quantity of DNA per saliva-stained punch was the Buccal DNA Collector (Bode) with a value of 2.83 ng. Two outlier results were present for the saliva-stained punches. Blood Direct Card #1 and the ProPrime Direct card (Fitzco, Inc.) yielded quantitative values of 0.286 ng DNA and 0.295 ng DNA, respectively.

### Performance of amplification kits

**Figure 1:**
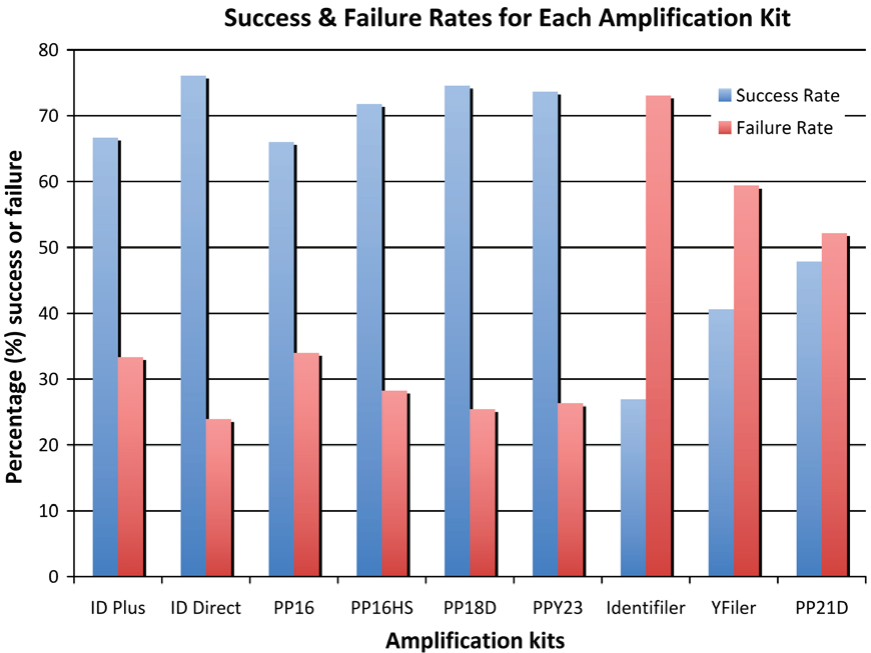
The calculated success and failure rates for each polymerase chain reaction amplification kit. Six amplification kits had success rates that exceeded 60%, while three amplification kits performed with success rates below 50%.

The performance of each PCR amplification kit was determined by calculating the number of complete and partial DNA profiles generated, followed by calculation of their respective success and failure rates as indicated in Figure 1. This summary takes into consideration approximately 550 amplifications, and included all ten substrates and both body fluids.

The application of a direct amplification protocol was most successful with AmpFlSTR® Identifiler® Plus and Direct PCR amplification kits, and PowerPlex® 16, 16 HS, 18D, and Y23 Systems. These six amplification kits yielded the highest number of complete DNA profiles for each substrate and body fluid with success rates greater than 60%.

The remaining three PCR amplification kits that were least successful following a direct amplification protocol were AmpFlSTR® Identifiler® and Yfiler, and PowerPlex® 21 System. These three amplification kits generated the fewest complete DNA profiles for each substrate and body fluid with success rates ranging between 27 to 48%. The lack of success in following a direct amplification protocol was not surprising for the two standard amplification kits from Applied Biosystems, since these kits are not recommended for direct amplification. Somewhat more surprising was the performance of the PowerPlex® 21 System. PowerPlex® 21 is a relatively new amplification kit intended for direct amplification. However, the technical manual does recommend pretreatment with PunchSolution^TM^ Kit (Promega Corporation), a step that was intentionally not included in this research project (9).

### Performance of substrates

Success rates for each substrate were calculated and compared to one another to determine which substrates performed best following direct amplification. Performance was based upon the number of complete or partial profiles generated by each substrate containing either blood or saliva. Comparison was also made between substrates with high success rates and PCR amplification kits with high success rates.

**Figure 2:**
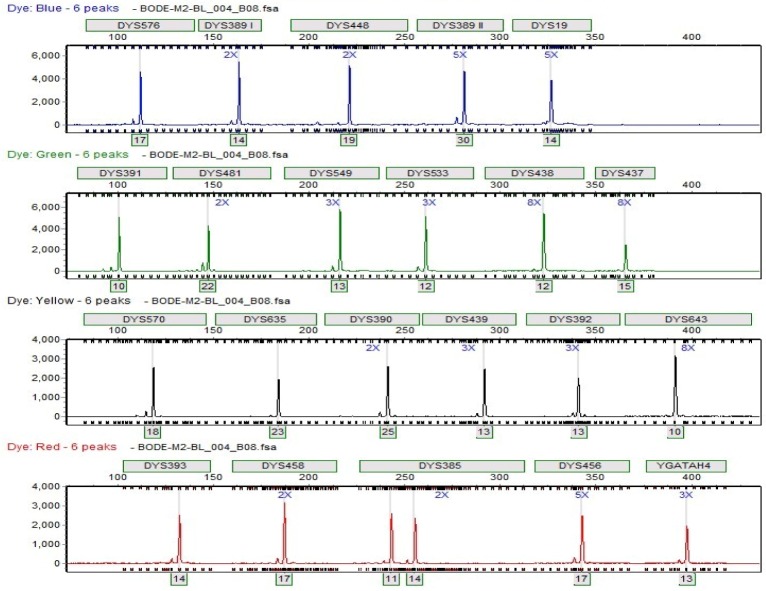
A complete Y-short tandem repeat (STR) profile generated from blood of a deceased man deposited on the Buccal DNA Collector (Bode). Polymerase chain reaction (PCR) amplification was performed with Promega PowerPlex® Y23 System.

**Figure 3:**
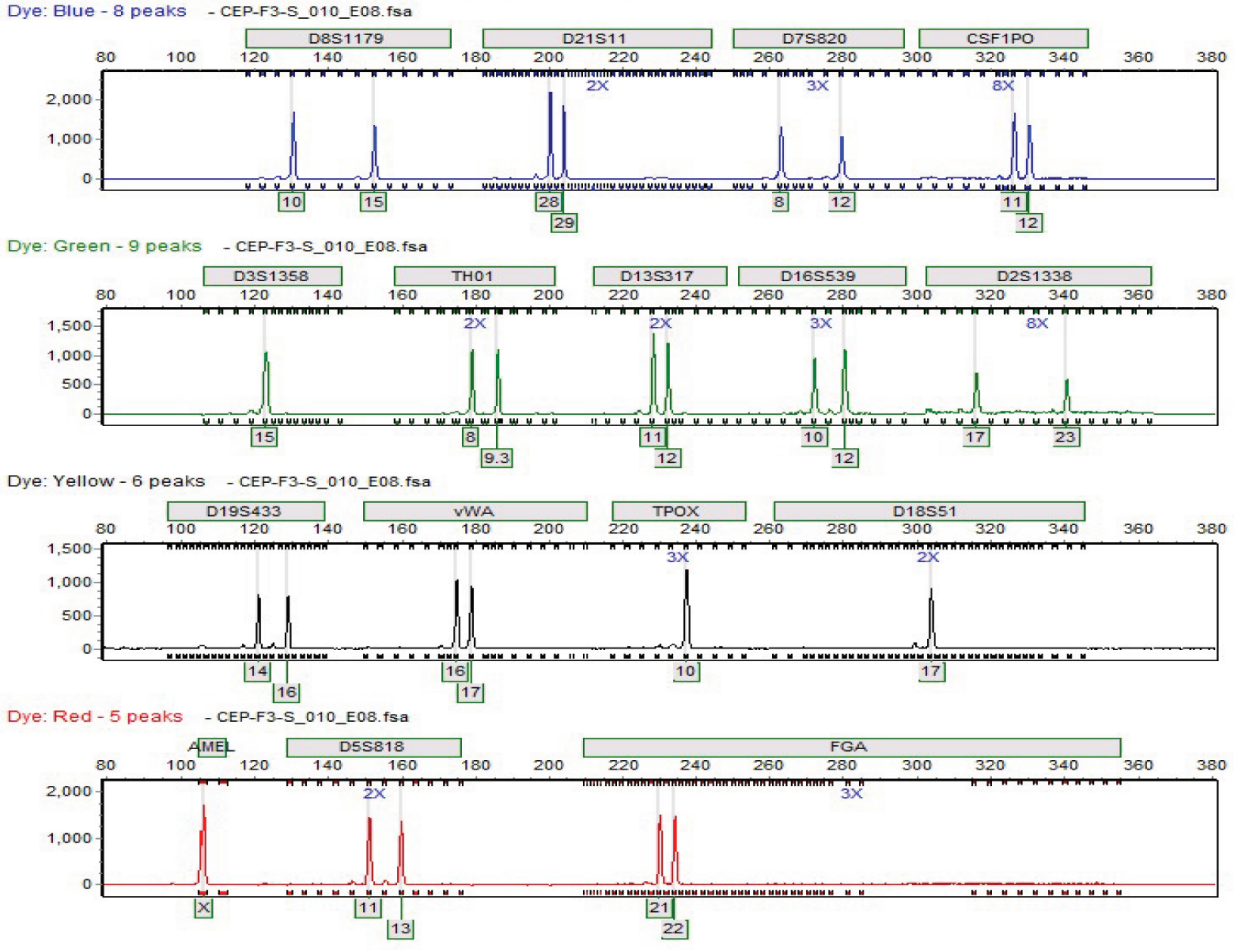
A complete autosomal short tandem repeat (STR) profile generated from saliva of a female donor deposited on the Fitzco CEP® Swab. Polymerase chain reaction amplification was performed with AmpFlSTR® Identifiler® PCR Amplification Kit.

When compared to the six amplification kits with the highest success rates, Blood Direct Card #1 (Fitzco, Inc.), Whatman EasiCollect (GE Healthcare Life Sciences), and the Buccal DNA Collector (Bode) all yielded the highest substrate success rates, 60% or higher. The CEP® swab (Fitzco, Inc.) also had a notably high success rate in combination with five out of the six successful amplification kits, and showed moderate success with the remaining amplification kits. The Buccal DNA Collector (Bode) and CEP® swab (Fitzco, Inc.) are untreated oral swabs. As shown in Figures 2 and 3, they yielded complete profiles even though they were not expected to yield as many concordant DNA profiles as other substrates. Blood Direct Card #1 (Fitzco, Inc.) also had a high success rate of approximately 71% with PowerPlex® 21 System (Promega Corporation). The Whatman EasiCollect sample storage device (GE Healthcare Life Sciences) performed similar to Blood Direct Card #1 (Fitzco, Inc.), however, it was not amplified with the male Y-STR amplification kits due to limited body fluid samples.

Sampling in various areas of each substrate and body fluid stain did not indicate significant differences in the robustness of the generated DNA profiles. This was found to be true for all ten substrates. No significant differences in the profiles generated were noted in body fluids amplified after 24 hours compared to body fluids amplified after approximately one year.

## Discussion

Evidence samples can sit for days to months prior to collection and subsequent analysis. Valuable studies have shown that drying time of body fluids does not have any effect on the success rate of a given amplification kit or the substrate (10).

Complete DNA profiles were successfully generated following a direct amplification protocol using both standard (non-direct) and direct PCR amplification kits. While six of the amplification kits and four of the paper substrates showed a higher level of performance, the remaining kits and substrates were able to generate complete DNA profiles, albeit at a lower success rate. The generation of complete DNA profiles appears to depend more on the success of the amplification kit rather than FTA®- or non-FTA®-based substrates. Generally, sample storage devices were more effective when paired with PCR amplification kits that were more successful in the application of a direct amplification protocol.

This procedure generates DNA profiles within a matter of hours from reference blood and saliva samples, showing great potential for use in forensic casework, data banking, and paternity laboratories. This method would be useful in forensic crime laboratories and greatly aid in the reduction of laboratory expenditures, and labor-intensive steps such as DNA extraction, purification, and quantification.
